# Neighborhood-Level Disparities in Hypertension Prevalence and Treatment Among Middle-Aged Adults

**DOI:** 10.1001/jamanetworkopen.2024.29764

**Published:** 2024-08-23

**Authors:** Madeleine M. Blazel, Adam T. Perzynski, Paul R. Gunsalus, Lyla Mourany, Douglas D. Gunzler, Robert W. Jones, Elizabeth R. Pfoh, Jarrod E. Dalton

**Affiliations:** 1 Cleveland Clinic Lerner College of Medicine of Case Western Reserve University, Cleveland, Ohio; 2 Center for Healthcare Research and Policy, Case Western Reserve University/MetroHealth Medical Center, Cleveland, Ohio; 3 Quantitative Health Sciences, Lerner Research Institute, Cleveland Clinic, Cleveland, Ohio; 4 Cleveland Clinic Value-Based Operations, Cleveland Clinic, Cleveland, Ohio; 5 Center for Value-Based Care Research, Cleveland Clinic, Cleveland, Ohio

## Abstract

**Importance:**

Hypertension in middle-aged adults (35-50 years) is associated with poorer health outcomes in late life. Understanding how hypertension varies by race and ethnicity across levels of neighborhood disadvantage may allow for better characterization of persistent disparities.

**Objective:**

To evaluate spatial patterns of hypertension diagnosis and treatment by neighborhood socioeconomic position and racial and ethnic composition.

**Design, Setting, and Participants:**

In this cross-sectional study of middle-aged adults in Cuyahoga County, Ohio, who encountered primary care in 2019, geocoded electronic health record data were linked to the area deprivation index (ADI), a neighborhood disadvantage measure, at the US Census Block Group level (ie, neighborhood). Neighborhoods were stratified by ADI quintiles, with the highest quintile indicating the most disadvantage. Data were analyzed between August 7, 2023, and June 1, 2024.

**Exposure:**

Essential hypertension.

**Main Outcomes and Measures:**

The primary outcome was a clinician diagnosis of essential hypertension. Spatial analysis was used to characterize neighborhood-level patterns of hypertension prevalence and treatment. Interaction analysis was used to compare hypertension prevalence by racial and ethnic group within similar ADI quintiles.

**Results:**

A total of 56 387 adults (median [IQR] age, 43.1 [39.1-46.9] years; 59.8% female) across 1157 neighborhoods, which comprised 3.4% Asian, 31.1% Black, 5.5% Hispanic, and 60.0% White patients, were analyzed. A gradient of hypertension prevalence across ADI quintiles was observed, with the highest vs lowest ADI quintile neighborhoods having a higher hypertension rate (50.7% vs 25.5%) and a lower treatment rate (61.3% vs 64.5%). Of the 315 neighborhoods with predominantly Black (>75%) patient populations, 200 (63%) had a hypertension rate greater than 35% combined with a treatment rate of less than 70%; only 31 of 263 neighborhoods (11.8%) comprising 5% or less Black patient populations met this same criterion. Compared with a spatial model without covariates, inclusion of ADI and percentage of Black patients accounted for 91% of variation in hypertension diagnosis prevalence among men and 98% among women. Men had a higher prevalence of hypertension than women across race and ADI quintiles, but the association of ADI and hypertension risk was stronger in women. Sex prevalence differences were smallest between Black men and women, particularly in the highest ADI quintile (1689 [60.0%] and 2592 [56.0%], respectively).

**Conclusions and Relevance:**

These findings show an association between neighborhood deprivation and hypertension prevalence, with disparities observed particularly among Black patients, emphasizing a need for structural interventions to improve community health.

## Key Points

**Question:**

Are there disparities in hypertension burden and treatment across neighborhoods by socioeconomic disadvantage and racial and ethnic composition?

**Findings:**

In this cross-sectional study of geocoded electronic health record data for 56 387 middle-aged adults, a disproportionate burden of hypertension prevalence and treatment was found in socioeconomically disadvantaged and predominantly Black neighborhoods.

**Meaning:**

These findings suggest the presence of neighborhood-level disparities in hypertension and treatment, indicating a need to investigate how to address these disparities at a structural level.

## Teaser

This cross-sectional study evaluates whether spatial patterns of hypertension among adults in midlife are associated with neighborhood disadvantage and racial and ethnic composition.

## Introduction

More than 116 million US adults have hypertension, which is the top modifiable individual-level risk factor for cardiovascular disease.^1,2,3^ A decrease in systolic blood pressure by 10 mm Hg is estimated to reduce the risk of a cardiovascular event by 20% to 30%.^3^ Middle age (35-50 years) is a critical time for intervention, as midlife hypertension has implications for poor cardiovascular health in subsequent decades^4^ and is associated with cognitive decline and dementia risk.^5,6^ The disproportionate burden of uncontrolled hypertension in non-Hispanic Black adults is a key contributor to existing disparities in stroke, cardiovascular disease, and mortality.^7,8^ Due to historical redlining, Black individuals have been systematically housed in neighborhoods that experienced disinvestment.^9^ Furthermore, where a person lives, including local resources and social environment, has been associated with hypertension risk.^10,11^

Critically, place-based interventions have shown positive outcomes and are necessary to target existing health inequities.^12,13,14^ Many reports of hypertension prevalence use national databases, such as the National Health and Nutrition Examination Survey (NHANES), that provide nationally representative estimates of disease at the population level.^15,16,17^ Few studies have reported small area–level hypertension rates,^4,18^ and none have evaluated to what extent neighborhoods connect the association between race and ethnicity and hypertension among midlife adults. In this report, we evaluate whether spatial patterns of hypertension diagnosis and treatment are associated with neighborhood socioeconomic position and racial and ethnic composition.

## Methods

### Data Sources and Inclusion Criteria

In this cross-sectional study, we analyzed electronic health record (EHR) data of adults aged 35 to 50 years who resided in Cuyahoga County, Ohio, and had 1 or more primary care appointments within the Cleveland Clinic Health System or MetroHealth System in 2019. The first primary care appointment attended in 2019 for each patient was classified as their index visit. The study was approved by the Cleveland Clinic Institutional Review Board (No. 22-896). Informed consent was waived due to institutional review board determination of minimal risk and that the research could not practicably be performed otherwise. Our report follows the Strengthening the Reporting of Observational Studies in Epidemiology (STROBE) reporting guideline.

We derived area deprivation index (ADI) values in Ohio from 2015 to 2019 American Community Survey 5-year data at the US Census Block Group level using the R package sociome.^19^ The ADI includes measures of income, education, housing, and occupation on a scale of 40 to 160, where a higher score indicates greater disadvantage. We used a local representation of the ADI due to technical limitations of the University of Wisconsin Neighborhood Atlas, described elsewhere^20,21^ (eFigure in Supplement 1).

### Study Outcomes and Variables

Our primary outcome was a clinician diagnosis of essential hypertension on or prior to the index visit. We defined essential hypertension as at least 1 *International Statistical Classification of Diseases and Related Problems, Tenth Revision* code in the Clinical Classification Software 98 diagnostic group. Our secondary outcome was hypertension treatment, which we defined as an antihypertensive medication prescribed on or up to 365 days prior to the index visit among patients with a hypertension diagnosis. In neighborhood-level analyses, we used the variable treatment rate, or the percentage of patients per neighborhood with hypertension who were prescribed an antihypertensive medication.

We grouped patients into neighborhoods based on their address at the time of their primary care visit. We then created ADI quintiles derived from all Ohio census block groups (ie, neighborhoods). We calculated the percentage of Black patients residing in each neighborhood and performed analyses using categories comparable with observed quartiles of our study sample within Cuyahoga County (≤5%, 5.1%-25.0%, 25.1%-75.0%, and >75.0%). This variable was included in models as the percentage of Black patients.

We obtained patient age, sex, and race and ethnicity from the EHR. Patients self-reported race as American Indian or Alaska Native, Asian, Black or African American, Native Hawaiian or Other Pacific Islander, White, or multiracial and self-reported ethnicity as Hispanic or not Hispanic. Due to sample size limitations, we analyzed the following combined racial and ethnic categories: Asian, Hispanic, non-Hispanic Black (hereafter Black), and non-Hispanic White (hereafter White). We excluded patients who identified as American Indian or Alaska Native, Native Hawaiian or Other Pacific Islander, and multiracial as well as patients with missing race and ethnicity, sex, or geographic identifiers.

To characterize patient health, we obtained body mass index and common comorbidities documented on or before the patients’ index visit. We considered patients to have a comorbidity if they had at least 1 *International Statistical Classification of Diseases, Tenth Revision* code for each of the following diseases: type 2 diabetes, lipid or metabolic disorders, coronary artery disease, chronic kidney disease, cerebrovascular disease, depression disorders, anxiety disorders, tobacco use, alcohol use, and substance abuse.

### Statistical Analysis

We descriptively compared patient demographics, comorbidities, and antihypertensive prescribing across ADI quintiles using frequencies and percentages for categorical variables and medians and IQRs for continuous variables. We estimated the prevalence of essential hypertension in middle-aged adults by ADI quintile and race and ethnicity and stratified results by sex because hypertension prevalence rates differ across men and women.^22^ As a sensitivity analysis, we used the ADI from Kind and Buckingham^23^ via the 2015 Wisconsin Neighborhood Atlas. The statistical analysis for this report was conducted between August 7, 2023, and June 1, 2024.

#### Spatial Analysis

We conducted a spatial analysis for a deeper understanding of neighborhood-level patterns of hypertension prevalence and treatment. We developed map visualizations to compare hypertension prevalence with the percentage of Black patients across neighborhoods, ADI, and antihypertensive medication prescribing rates among those with hypertension. We estimated area-level correlation measures using Pearson correlation coefficients and Moran *I* statistics to identify the strength of the association among our variables of interest.

To characterize neighborhood-level hypertension rates while accounting for potential spatial correlation between neighboring block groups, we used sex-stratified bayesian conditional autoregressive (CAR) Poisson rate models. We developed 3 models: (1) a null model with no covariates (ie, random and spatial effects only), (2) a model accounting for ADI quintile to characterize hypertension prevalence across socioeconomic position, and (3) a model accounting for the interaction of ADI quintile and the percentage of Black patients per neighborhood to understand the overall degree of neighborhood-level variability in hypertension accounted for by these 2 factors.

#### Interaction Analysis

We developed an interaction model to compare hypertension prevalence by racial and ethnic group within similar ADI quintiles. We conducted a multivariable logistic regression with a 3-way interaction among sex, race and ethnicity, and ADI quintile. Interaction terms were selected a priori to investigate the heterogeneity of hypertension across racial and ethnic groups in each ADI quintile, stratified by sex.^22^ Odds ratios (ORs) from this model are displayed with the hypertension prevalence of each subgroup analyzed.

All analyses were performed using R, version 4.3.1 statistical software (R Foundation for Statistical Computing) within the Posit Workbench–integrated development environment, version 2023.09.0 + 463 (Posit Software, PBC). Bayesian estimates are reported as posterior mean (95% credible interval [CrI]), and frequentist estimates are reported as maximum likelihood estimate (95% CI).

## Results

A total of 60 546 patients met the inclusion criteria. We removed 3130 unique patients due to missing race or ethnicity (n = 3120), missing sex (n = 4), or addresses that could not be geocoded to a US Census Block Group (n = 7). We removed 1029 patients in race categories with small sample sizes (113 American Indian or Alaska Native, 27 Native Hawaiian or Pacific Islander, and 889 multiracial) (eTable 1 in Supplement 1). Our final analytic sample included 56 387 adults who resided in 1157 Cuyahoga County neighborhoods (block groups). The number of neighborhoods per quintile was as follows: quintile 1, 259; quintile 2, 176; quintile 3, 159; quintile 4, 187; and quintile 5, 376.

### Descriptive Analysis

Among the 56 387 patients analyzed, the median (IQR) age was 43.1 (39.1-46.9) years, 59.8% were female, and 40.2% were male. Overall, 21.6% of patients lived in the highest (least resources) ADI quintile, and 31.2% lived in the lowest (most resources) ADI quintile. The cohort racial and ethnic background included, 1944 Asian (3.4%), 17 557 Black (31.1%), 3089 Hispanic (5.5%), and 33 797 White (60.0%) patients. The racial background of the population differed across quintiles; in the lowest ADI quintile, 1229 patients (7.0%) were Black and 15 146 (86.1%) were White compared with 7436 Black patients (61.2%) and 3006 White patients (24.6%) in the highest ADI quintile. We found a socioeconomic gradient for most comorbidities, including cerebrovascular disease, obesity, and coronary artery disease. Patients residing in neighborhoods in the highest ADI quintile had a higher prevalence of hypertension (50.7% vs 25.5%) and lower treatment rates (61.3% vs 64.5%) (Table 1).

**Table 1.** Demographic and Clinical Variables by ADI Quintile (N = 56 387)

Variable	ADI quintile, No. (%)				
	Quintile 1 (n = 17 586)	Quintile 2 (n = 9263)	Quintile 3 (n = 8201)	Quintile 4 (n = 9137)	Quintile 5 (n = 12 200)
**Demographic**					
Age, median (IQR), y	43.2 (39.3-46.9)	42.7 (38.8-46.7)	42.9 (39.0-46.9)	43.1 (39.1-46.9)	43.3 (39.3-47.1)
Sex					
Female	10 121 (57.6)	5524 (59.6)	4944 (60.3)	5608 (61.4)	7504 (61.5)
Male	7465 (42.4)	3739 (40.4)	3257 (39.7)	3529 (38.6)	4696 (38.5)
Race and ethnicity					
Asian	1021 (5.8)	394 (4.3)	203 (2.5)	192 (2.1)	134 (1.1)
Black	1229 (7.0)	1775 (19.2)	2572 (31.4)	4518 (49.4)	7463 (61.2)
Hispanic	190 (1.1)	291 (3.1)	295 (3.6)	716 (7.8)	1597 (13.1)
White	15 146 (86.1)	6803 (73.4)	5131 (62.6)	3711 (40.6)	3006 (24.6)
**Clinical**					
Hypertension					
Diagnosis	4481 (25.5)	3042 (32.8)	3157 (38.5)	3997 (43.7)	6186 (50.7)
Prescribed antihypertensive medication (n = 20 863)^a^	2892 (64.5)	1962 (64.5)	2106 (66.7)	2583 (64.6)	3794 (61.3)
Comorbidities					
Coronary artery disease	392 (2.2)	265 (2.9)	287 (3.5)	381 (4.2)	723 (5.9)
Chronic kidney disease	275 (1.6)	188 (2.0)	202 (2.5)	322 (3.5)	610 (5.0)
Cerebrovascular disease	213 (1.2)	175 (1.9)	201 (2.5)	262 (2.9)	528 (4.3)
Type 2 diabetes	1013 (5.8)	915 (9.9)	1034 (12.6)	1466 (16.0)	2593 (21.3)
Obesity (BMI >30)	6168 (35.1)	4446 (48.0)	4513 (55.0)	5538 (60.6)	7598 (62.3)
Lipid or metabolic disorder	6675 (38.0)	3482 (37.6)	3250 (39.6)	3700 (40.5)	5129 (42.0)
Depression	4480 (25.5)	2899 (31.3)	2820 (34.4)	3240 (35.5)	4945 (40.5)
Anxiety	6230 (35.4)	3562 (38.5)	3062 (37.3)	3147 (34.4)	4042 (33.1)
Alcohol use disorder	705 (4.0)	514 (5.5)	564 (6.9)	806 (8.8)	1622 (13.3)
Substance use disorder	2197 (12.5)	1891 (20.4)	2169 (26.4)	2925 (32.0)	5160 (42.3)
Tobacco use disorder	1849 (10.5)	1642 (17.7)	1916 (23.4)	2568 (28.1)	4578 (37.5)
Census block groups, No.	259	176	159	187	376

Abbreviations: ADI, area deprivation index; BMI, body mass index (calculated as weight in kilograms divided by height in meters squared).

^a^ Reported percentages of antihypertensive prescription were limited to the population with a current hypertension diagnosis.

We observed a gradient in hypertension prevalence across ADI quintiles for almost all racial and ethnic groups (Table 2). Across ADI quintiles, men consistently had higher rates of hypertension than women, though prevalence differences were smallest between Black men and women, particularly in the highest ADI quintile (1689 of 2833 [65.0%] and 2592 of 4630 [56.0%], respectively). For all quintiles combined, Black men and women had the highest prevalence of hypertension compared with all other racial and ethnic groups (men, 3644 of 6446 [56.5%]; women, 5715 of 11 111 [51.4%]).

**Table 2.** Hypertension Prevalence and Odds of Hypertension Diagnosis Derived From Multivariable Logistic Regression With Interaction Among Sex, Race, and ADI Quintile

ADI quintile by race and ethnicity	Male				Female			
	Total population	**Prevalence, No. (%)**	OR (95% CI)	*P* value	Total population	Prevalence, No. (%)	OR (95% CI)	*P* value
Asian								
Quintile 1	376	86 (22.9)	1 [Reference]	NA	645	76 (11.8)	1 [Reference]	NA
Quintile 2	170	46 (27.1)	1.25 (0.82-1.89)	.29	224	39 (17.4)	1.58 (1.03-2.39)	.03
Quintile 3	94	24 (25.5)	1.16 (0.68-1.93)	.59	109	22 (20.2)	1.89 (1.10-3.16)	.02
Quintile 4	80	20 (25.0)	1.12 (0.63-1.94)	.68	112	24 (21.4)	2.04 (1.21-3.36)	.006
Quintile 5	59	22 (37.3)	2.01 (1.11-3.56)	.02	75	14 (18.7)	1.72 (0.89-3.14)	.09
Black								
Quintile 1	469	236 (50.3)	1 [Reference]	NA	760	328 (43.2)	1 [Reference]	NA
Quintile 2	609	323 (53.0)	1.12 (0.88-1.42)	.38	1166	514 (44.1)	1.04 (0.86-1.25)	.69
Quintile 3	892	481 (53.9)	1.16 (0.92-1.45)	.21	1680	813 (48.4)	1.24 (1.04-1.47)	.02
Quintile 4	1643	915 (55.7)	1.24 (1.01-1.52)	.04	2875	1468 (51.1)	1.37 (1.17-1.62)	<.001
Quintile 5	2833	1689 (59.6)	1.46 (1.20-1.77)	<.001	4630	2592 (56.0)	1.68 (1.44-1.96)	<.001
Hispanic								
Quintile 1	70	23 (32.9)	1 [Reference]	NA	120	21 (17.5)	1 [Reference]	NA
Quintile 2	88	38 (43.2)	1.55 (0.81-3.01)	.19	203	67 (33.0)	2.32 (1.35-4.12)	.003
Quintile 3	108	50 (46.3)	1.76 (0.95-3.33)	.08	187	55 (29.4)	1.96 (1.13-3.52)	.02
Quintile 4	249	102 (41.0)	1.42 (0.82-2.51)	.22	467	149 (31.9)	2.21 (1.35-3.76)	.002
Quintile 5	560	242 (43.2)	1.56 (0.93-2.67)	.10	1037	358 (34.5)	2.49 (1.56-4.15)	<.001
White								
Quintile 1	6550	2103 (32.1)	1 [Reference]	NA	8596	1608 (18.7)	1 [Reference]	NA
Quintile 2	2872	1042 (36.3)	1.20 (1.10-1.32)	<.001	3931	973 (24.8)	1.43 (1.31-1.56)	<.001
Quintile 3	2163	877 (40.5)	1.44 (1.30-1.59)	<.001	2968	835 (28.1)	1.70 (1.54-1.87)	<.001
Quintile 4	1557	638 (41.0)	1.47 (1.31-1.64)	<.001	2154	681 (31.6)	2.01 (1.81-2.23)	<.001
Quintile 5	1244	567 (45.6)	1.77 (1.57-2.00)	<.001	1762	702 (49.8)	2.88 (2.58-3.21)	<.001

Abbreviations: ADI, area deprivation index; NA, not applicable; OR, odds ratio.

### Spatial Analysis

We found a high degree of spatial clustering for hypertension rates (Moran *I* = 0.58; *P* < .001) and a small but significant degree of spatial clustering for antihypertensive prescribing (Moran *I* = 0.05; *P* = .002). Higher neighborhood-level prevalence of hypertension was correlated with a higher ADI quintile (*r* = 0.73; *P* < .001) (Figure, A) and a higher percentage of Black patients (*r* = 0.64; *P* < .001) (Figure, B). Neighborhoods with a greater percentage of Black patients tended to have a higher ADI score (*r* = 0.62; *P* < .001). We assessed how groups of neighborhoods compared with national hypertension prevalence (33% based on a cutoff of 140/90 mm Hg) and treatment prevalence (73%) estimated using NHANES data by Aggarwal et al.^24^ Among the 315 neighborhoods with predominantly Black (>75%) patient populations, 200 neighborhoods (63%) had hypertension rates of greater than 35% combined with antihypertensive prescription rates of less than 70% (Figure, C). Of those 200 neighborhoods, 80% were in the highest ADI quintile. In comparison, only 31 of the 263 neighborhoods (11.8%) in which Black patients comprised 5% or less of the population had hypertension rates of greater than 35% combined with treatment rates of less than 70%.

**Figure.**
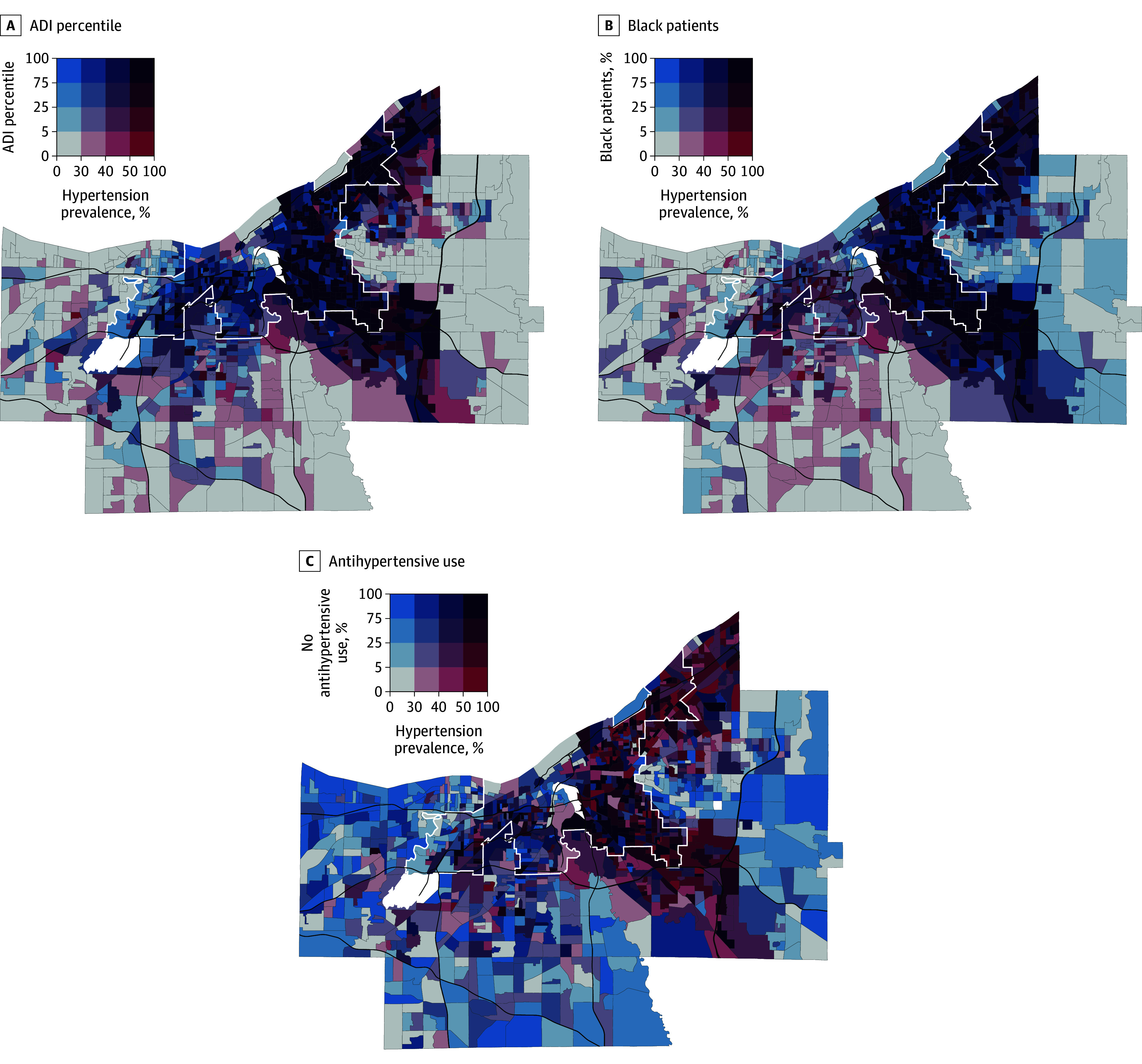
Hypertension Diagnosis in Midlife A and B, Included are 56 387 residents across 1157 neighborhoods. C, The sample was limited to the 20 863 patients with diagnosed hypertension. Cut points for antihypertensive use and percentages of blood pressure control and Black patients were comparable with observed quartiles within Cuyahoga County, Ohio. White lines demarcate Cleveland city limits, and black lines denote major highways. White shaded areas have sparse or no residential population (ie, airports, industrial districts). ADI indicates area deprivation index.

In the CAR Poisson rate model incorporating ADI quintile (model 1), men living in neighborhoods in the highest ADI quintile had a 58% increased prevalence of hypertension compared with the lowest ADI quintile (posterior mean, 1.58; 95% CrI, 1.46-1.70) (Table 3). In the women’s CAR model incorporating ADI quintile only, neighborhoods in the highest quintile had twice the prevalence of hypertension compared with the lowest ADI quintile (posterior mean, 2.08; 95% CrI, 1.91-2.25). Compared with a null CAR model (no covariates), ADI quintile accounted for 85% of neighborhood-level variation in men and 78% in women. The CAR model incorporating an interaction between ADI quintile and percentage of Black patients per neighborhood accounted for 91% of spatial variation in hypertension prevalence in men and 98% in women compared with the null model (performance characteristics shown in eTable 2 in Supplement 1).

**Table 3.** Hypertension Prevalence Ratios Derived From Sex-Stratified Conditional Autoregressive Poisson Rate Models Incorporating ADI Quintile Associated With Patients’ Census Block Group of Residence

ADI quintile	Posterior mean (95% CrI)	
	Male	Female
Quntile 1	1 [Reference]	1 [Reference]
Quntile 2	1.19 (1.11-1.28)	1.40 (1.30-1.51)
Quntile 3	1.31 (1.22-1.42)	1.59 (1.47-1.72)
Quntile 4	1.39 (1.29-1.50)	1.79 (1.65-1.93)
Quntile 5	1.58 (1.46-1.70)	2.08 (1.91-2.25)

Abbreviations: ADI, area deprivation index; CrI, credible interval.

### Interaction Analysis

In our interaction model, which included a 3-way interaction among sex, race and ethnicity, and ADI quintile, the odds of hypertension in the highest vs lowest ADI quintile were higher for White men (OR, 1.77; 95% CI, 1.57-2.00; *P* < .001) and White women (OR, 2.88; 95% CI, 2.58-3.21; *P* < .001) compared with Black men (OR. 1.46; 95% CI, 1.20-1.77; *P* < .001) and Black women (OR, 1.68; 95% CI, 1.44-1.96; *P* < .001) (Table 2). Hispanic women had significantly increased odds of hypertension with increasing neighborhood disadvantage (quintile 5 vs quintile 1: OR, 2.49; 95% CI, 1.56-4.15; *P* < .001), while higher ADI quintiles were comparatively not associated with higher odds of hypertension within Hispanic men. Asian women had relatively smaller (compared with other women) but significant increases in hypertension odds across most ADI quintiles (quintile 2: OR, 1.58 [95% CI, 1.03-2.39; *P* = .03]; quintile 3: OR, 1.89 [95% CI, 1.10-3.16; *P* = .02]; quintile 4: OR, 2.04 [95% CI, 1.21-3.36; *P* = .006]). Among Asian men, we found increased odds of hypertension only for patients in the highest ADI quintile neighborhoods compared with those in the lowest ADI quintile neighborhoods (OR, 2.01; 95% CI, 1.11-3.56; *P* = .02).

### Sensitivity Analysis

We conducted a sensitivity analysis of our spatial analysis and interaction analysis using ADI quintiles from the 2015 Wisconsin Neighborhood Atlas.^23^ We excluded 99 patients who resided in a block group with suppressed ADI due to a high group quarter population. Results of the spatial analysis were similar overall (eTable 3 in Supplement 1). In the interaction analysis, ORs for hypertension were lower across Wisconsin Neighborhood Atlas–derived ADI quintiles for Hispanic women as in our primary analysis; results were otherwise comparable (eTable 4 in Supplement 1).

## Discussion

In this cross-sectional study, we found corresponding increases in hypertension prevalence as neighborhood disadvantage and the percentage of Black patients residing in a neighborhood increased. We identified a higher burden of midlife hypertension in Black adults compared with other racial and ethnic groups that persisted across levels of socioeconomic disadvantage. We also found that living in socioeconomically disadvantaged neighborhoods was associated with higher hypertension rates among people of all racial and ethnic backgrounds.

### Hypertension in Midlife

A growing body of evidence suggests that midlife hypertension increases the risk for heart failure, coronary heart disease, cognitive decline, and all-cause mortality.^5,6,25,26^ In alignment with prior epidemiologic research, we found that men had a greater prevalence of hypertension than women.^27,28,29^ However, the association of worsening neighborhood socioeconomic status and hypertension risk was more pronounced among Black, Hispanic, and White women. These findings are concordant with the existing literature, including a longitudinal cohort that showed the steepest annual growth in systolic blood pressure for women living in more socioeconomically vulnerable areas.^30,31^ Thus, given the long-term consequences for health and mortality, midlife is a key time for optimization of cardiovascular risk factors. To our knowledge, our study is the first to describe the composition of neighborhood-level disparities in hypertension prevalence and treatment using the intersection of racial and ethnic composition and socioeconomic position.

### Racial Residential Segregation

In the spatial analyses, interactions between ADI and the percentage of Black patients per neighborhood accounted for nearly all the spatial variation in hypertension rates, beyond that accounted for by ADI alone in our models. This finding aligns with the history of racial residential segregation—including 20th century redlining practices that systematically excluded Black individuals from housing opportunities^9^—in Cuyahoga County, Ohio, which remains one of the most segregated areas in the US. In this study, we conceptualized race and ethnicity as socially constructed variables representing exposure to racism at interpersonal and structural levels.^9,32,33,34^ In a robust regional population similar to our cohort, measures of structural racism were associated with a higher burden of hypertension and other chronic conditions.^35^ Our stratified analysis revealed that White patients who lived in the highest ADI quintile were also diagnosed with hypertension at high rates. This finding suggests that neighborhood disinvestment and economic decline may be associated with health measures of all who live there, regardless of their racial and ethnic background.

Importantly, we found significant treatment disparities among neighborhoods that geographically corresponded to patterns of historical racial residential segregation and neighborhood disadvantage.^36^ There were lower antihypertensive medication treatment rates within socioeconomically disadvantaged, predominately Black neighborhoods than in more resourced neighborhoods, suggesting that national estimates may mask nuanced variation in treatment across small areas. Despite reported national disparities in hypertension prevalence, previous estimates have also found that antihypertensive treatment rates for Black individuals in the US are comparable with those for White individuals.^8,24,27,37^ Yet, at the neighborhood level, treatment varies across neighborhood resource level and racial and ethnic composition, suggesting that localized disparities persist.

### Systemic Racism

Ongoing systemic racism has been independently associated with an increased risk of high blood pressure for both Black and Hispanic individuals.^38^ In a prior cross-sectional analysis using NHANES and US Census data, Black adults had higher odds of hypertension regardless of individual or neighborhood poverty level, while only White adults living in low-income neighborhoods had higher odds of hypertension.^17,35^ In Cuyahoga County, we report that Black men and women in midlife have hypertension prevalence rates of 57% and 51%, respectively, which are comparable with the highest national estimates within Black adults, depending on the definitions used.^7,24,39^ In our analysis, nearly two-thirds of predominately (>75% population) Black neighborhoods in Cuyahoga County simultaneously exceeded the national average hypertension prevalence and were below average in antihypertensive treatment rates. Furthermore, 80% of these neighborhoods were characterized by a lack of socioeconomic resources. Hence, our study shows an intersectionality of race and place in the context of disparities in midlife hypertension, which is a critical factor in determining health and longevity later in the life course.

### Structural Interventions

Our findings extend prior research on the association between neighborhoods and hypertension outcomes.^40,41^ Successful place-based efforts have included using barber shops and salons to screen patients for hypertension who might otherwise not access primary care.^42^ Place-based interventions that are more ecologically focused include setting up farmers’ markets and attracting grocery stores to food deserts to support access to healthy food.^11^

Larger-scale approaches have included improving housing to ensure that it is free from lead (a known risk factor for hypertension).^43^ Access to safe housing may reduce stress, another risk factor for hypertension.^44,45^ Health system interventions, such as screening for and addressing health-related social needs that are inequitably distributed across neighborhoods, may improve blood pressure control, as well as large-scale hypertension-focused quality improvement programs. Most health system efforts are focused on the individual patient care setting^46^ and are effective overall, but they do not address the disparities in blood pressure control across diverse patient populations.^47,48^ The utility of our approach is not only that we used the ADI—a measure of income, education, housing, and employment resources, which are related to place-based interventions—but also that we demonstrate in the Figure how spatial analysis could be used to identify specific neighborhoods in which to place these interventions.

### Limitations

Our study has several limitations. Our population is limited to patients who interact with the health care system and obtain primary care services. Thus, the hypertension estimates provided are not the true neighborhood-level prevalence of hypertension, as only individuals who can access care are represented. It is also possible that patients may have had undiagnosed hypertension, thus making the prevalence estimates more conservative than the true estimates. We were also unable to determine whether the patients who had been prescribed an antihypertensive medication were taking the medication to treat hypertension, since antihypertensive medications are a broad category and are used to treat other conditions. Other indications for antihypertensive medication may have resulted in an overestimate of the number of patients being treated pharmacologically. Additionally, while race and ethnicity in the EHR are intended to be self-reported, we cannot exclude the possibility that this information was documented by another party without adequate verification. Finally, we focused on a single county for our analysis, with recognized limitations in generalizability of findings. However, the redlining and downstream structural racism that created the residential segregation in Cuyahoga County are widespread in the US.^9,49^ Since racial segregation among neighborhoods is found in many large US cities, further research should investigate place-based disparities to promote equitable hypertension care in other locales.

## Conclusions

The findings of this cross-sectional study suggest stark racial and neighborhood disparities in hypertension prevalence and antihypertensive treatment among adults in midlife, with a significant burden of undertreated hypertension in socioeconomically disadvantaged and racially segregated communities. Using spatial analysis techniques to identify neighborhoods in need, future research might investigate structural interventions to address place-based hypertension disparities.

## Notes

Supplement 1. **eTable 1.** Demographic, Neighborhood, and Clinical Variables Between the Analyzed Cohort and Cohorts Excluded for Missing Data or Limited Sample Sizes of Self-Reported Race Categories **eTable 2.** Performance Characteristics of Sex-Stratified Conditional Autoregressive Poisson Regressions **eTable 3.** Sensitivity Analysis of Hypertension Prevalence and Odds Ratios (ORs) of Hypertension Diagnosis Derived From Multivariable Logistic Regression With Interaction Among Sex, Race, and ADI Quintile Derived From the Wisconsin Neighborhood Atlas **eTable 4.** Sensitivity Analysis of Hypertension Prevalence Ratios Derived From Sex-Stratified Conditional Autoregressive (CAR) Poisson Rate Models Incorporating ADI Quintile Associated With Patients’ Census Block Group of Residence **eFigure.** ADI Deciles for US Census Block Groups in Cuyahoga County, Ohio, Derived From Sociome and Neighborhood Atlas

Supplement 2.
Data Sharing Statement
