# Supplementary material for: Neighborhood-Level Disparities in Hypertension Prevalence and Treatment Among Middle-Aged Adults
Source: JAMA Netw Open. 2024 Aug 23;7(8):e2429764. doi: 10.1001/jamanetworkopen.2024.29764 (PMC11344236; doi:10.1001/jamanetworkopen.2024.29764)
Supplement: Supplement 1. — eTable 1. Demographic, Neighborhood, and Clinical Variables Between the Analyzed Cohort and Cohorts Excluded for Missing Data or Limited Sample Sizes of Self-Reported Race Categories eTable 2. Performance Characteristics of Sex-Stratified Conditional Autoregressive Poisson Regressions eTable 3. Sensitivity Analysis of Hypertension Prevalence and Odds Ratios (ORs) of Hypertension Diagnosis Derived From Multivariable Logistic Regression With Interaction Among Sex, Race, and ADI Quintile Derived From the Wisconsin Neighborhood Atlas eTable 4. Sensitivity Analysis of Hypertension Prevalence Ratios Derived From Sex-Stratified Conditional Autoregressive (CAR) Poisson Rate Models Incorporating ADI Quintile Associated With Patients’ Census Block Group of Residence eFigure. ADI Deciles for US Census Block Groups in Cuyahoga County, Ohio, Derived From Sociome and Neighborhood Atlas [file jamanetwopen-e2429764-s001.pdf]

Supplemental Online Content

Blazel MM, Perzynski AT, Gunsalus P, et al. Neighborhood-level disparities in hypertension prevalence and treatment among middle-aged adults. *JAMA Netw Open*. 2024;7(8):e2429764. doi:10.1001/jamanetworkopen.2024.29764

eTable 1. Demographic, Neighborhood, and Clinical Variables Between the Analyzed Cohort and Cohorts Excluded for Missing Data or Limited Sample Sizes of Self-Reported Race Categories

eTable 2. Performance Characteristics of Sex-Stratified Conditional Autoregressive Poisson Regressions

eTable 3. Sensitivity Analysis of Hypertension Prevalence and Odds Ratios (ORs) of Hypertension Diagnosis Derived From Multivariable Logistic Regression With Interaction Among Sex, Race, and ADI Quintile Derived From the Wisconsin Neighborhood Atlas

eTable 4. Sensitivity Analysis of Hypertension Prevalence Ratios Derived From Sex-Stratified Conditional Autoregressive (CAR) Poisson Rate Models Incorporating ADI Quintile Associated With Patients' Census Block Group of Residence

eFigure. ADI Deciles for US Census Block Groups in Cuyahoga County, Ohio, Derived From Sociome and Neighborhood Atlas

This supplemental material has been provided by the authors to give readers additional information about their work.

© 2024 Blazel MM et al. *JAMA Network Open*.

eTable 1. Demographic, Neighborhood, and Clinical Variables Between the Analyzed Cohort and Cohorts Excluded for Missing Data or Limited Sample Sizes of Self-Reported Race Categories. Results are presented as median [IQR] for continuous variables and count (percentage) for categorical variables. Reported percentages of antihypertensive prescription were limited to the population with a current hypertension diagnosis.

	**Analyzed cohort (n=56387)**	**Excluded patients (n=4159)**	
		**Missing race, sex, or ADI (n=3130)**	**Small sample size of racial identity (n=1029)**
**Demographics and Neighborhood Variables (No. (%))**			
Age (median [IQR])	43.1 [39.1, 46.9]	43.0 [39.1, 46.7]	42.3 [38.4, 46.1]
Female	33701 (60)	*[n=3126]* 1639 (52)	594 (58)
Race and ethnicity		*[n=11]*	
Asian	1944 (3.4)	0	0
Hispanic	3089 (5.5)	1 (9.1)	0
Non-Hispanic Black	17557 (31)	3 (27)	0
Non-Hispanic White	33797 (60)	7 (64)	0
American Indian/Alaska	0	0	113 (11)
Native	0	0	889 (86)
Multiracial	0	0	27 (2.6)
Native Hawaiian/Pacific			
Islander			
ADI Quintile		*[n=3123]*	
Q1	17586 (31)	1135 (36)	341 (33)
Q2	9263 (16)	576 (18)	221 (21)
Q3	8201 (15)	451 (14)	155 (15)
Q4	9137 (16)	463 (15)	146 (14)
Q5	12200 (22)	498 (16)	166 (16)
**Hypertension Variable**			
Hypertension diagnosis	20863 (37)	945 (30)	332 (32)
Prescribed antihypertensive prescription	*[n=20863]* 13337 (64)	*[n=945]* 614 (65)	*[n=332]* 216 (65)
**Comorbidities**			
Coronary artery disease	2048 (3.6)	89 (2.8)	42 (4.1)
Chronic kidney disease	1597 (2.8)	57 (1.8)	25 (2.4)
Cerebrovascular disease	1379 (2.4)	50 (1.6)	19 (1.8)
Type 2 diabetes mellitus	7021 (12)	344 (11)	146 (14)
Obesity (BMI > 30)	28263 (50)	1421 (45)	475 (46)
Lipid or metabolic disorder	22236 (39)	1257 (40)	405 (39)
Depression	18384 (33)	841 (27)	298 (29)
Anxiety	20043 (36)	998 (32)	307 (30)
Alcohol use disorder	4211 (7.5)	148 (4.7)	54 (5.2)
Substance use disorder	14342 (25)	512 (16)	228 (22)
Tobacco use disorder	12553 (22)	448 (14)	200 (19)

© 2024 Blazel MM et al. *JAMA Network Open*.

eTable 2. Performance Characteristics of Sex-Stratified Conditional Autoregressive Poisson Regressions

	Men			Women		
	**Null Model**	**Model 1**	**Model 2**	**Null Model**	**Model 1**	**Model 2**
**WAIC**	5249.68	5200.35	5171.79	5670.19	5554.39	5476.45
**Residual Moran Coefficient**	0.0402	0.0310	0.0154	0.0535	0.0476	0.0404

eTable 3. Sensitivity Analysis of Hypertension Prevalence and Odds Ratios (ORs) of Hypertension Diagnosis Derived From Multivariable Logistic Regression With Interaction Among Sex, Race, and ADI Quintile Derived From the Wisconsin Neighborhood Atlas

	Men				Women			
	**Asian**							
**ADI Quintile**	**Prevalence No. (%)**	**OR**	**CI**	**p-value**	**Prevalence No. (%)**	**OR**	**CI**	**p-value**
Q1	86 (22)	Ref	--	--	75 (11)	Ref	--	--
Q2	47 (27)	1.28	0.85, 1.93	0.24	35 (17)	1.55	0.99, 2.37	0.05
Q3	28 (27)	1.34	0.81, 2.19	0.25	31 (22)	2.14	1.33, 3.38	**0.001**
Q4	22 (30)	1.53	0.87, 2.64	0.13	17 (22)	2.23	1.20, 3.95	**0.008**
Q5	15 (43)	2.66	1.29, 5.40	**0.007**	16 (20)	1.96	1.05, 3.50	**0.03**
	**Hispanic**							
Q1	27 (39)	Ref	--	--	35 (27)	Ref	--	--
Q2	17 (30)	0.66	0.31, 1.38	0.28	28 (22)	0.77	0.43, 1.36	0.36
Q3	66 (47)	1.41	0.79, 2.55	0.26	81 (31)	1.25	0.79, 2.01	0.35
Q4	100 (40)	1.06	0.62, 1.84	0.84	141 (31)	1.22	0.80, 1.91	0.36
Q5	245 (44)	1.20	0.72, 2.02	0.48	365 (35)	1.49	1.00, 2.26	0.06
	**Non-Hispanic Black**							
Q1	228 (51)	Ref	--	--	331 (44)	Ref	--	--
Q2	278 (51)	1.00	0.78, 1.28	1.0	466 (45)	1.06	0.88, 1.28	0.56
Q3	472 (54)	1.13	0.90, 1.41	0.31	707 (47)	1.15	0.96, 1.37	0.12
Q4	945 (57)	1.25	1.01, 1.54	**0.04**	1585 (50)	1.32	1.12, 1.55	**0.001**
Q5	1712 (59)	1.38	1.13, 1.68	**0.002**	2620 (56)	1.67	1.43, 1.95	**<0.001**
	**Non-Hispanic White**							
Q1	1848 (31)	Ref	--	--	1397 (18)	Ref	--	--
Q2	1145 (37)	1.30	1.19, 1.43	**<0.001**	987 (24)	1.42	1.30, 1.56	**<0.001**
Q3	1014 (40)	1.48	1.35, 1.64	**<0.001**	949 (28)	1.76	1.60, 1.94	**<0.001**
Q4	659 (42)	1.63	1.45, 1.83	**<0.001**	755 (33)	2.26	2.03, 2.51	**<0.001**
Q5	551 (46)	1.86	1.64, 2.11	**<0.001**	703 (40)	3.07	2.74, 3.43	**<0.001**

© 2024 Blazel MM et al. *JAMA Network Open*.

eTable 4. Sensitivity Analysis of Hypertension Prevalence Ratios Derived From Sex-Stratified Conditional Autoregressive (CAR) Poisson Rate Models Incorporating ADI Quintile Associated With Patients' Census Block Group of Residence. Results are presented as posterior means and 95% credible intervals (CI).

	**Men**		**Women**	
	**Mean**	**95% CI**	**Mean**	**95% CI**
**ADI Quintile**				
Q1	Ref	--	Ref	--
Q2	1.22	1.14, 1.31	1.39	1.29, 1.50
Q3	1.37	1.28, 1.47	1.64	1.52, 1.78
Q4	1.49	1.38, 1.61	1.91	1.75, 2.07
Q5	1.65	1.53, 1.77	2.22	2.04, 2.41

© 2024 Blazel MM et al. *JAMA Network Open*.

eFigure. ADI Deciles for US Census Block Groups in Cuyahoga County, Ohio, Derived From Sociome and Neighborhood Atlas

A) Sociome

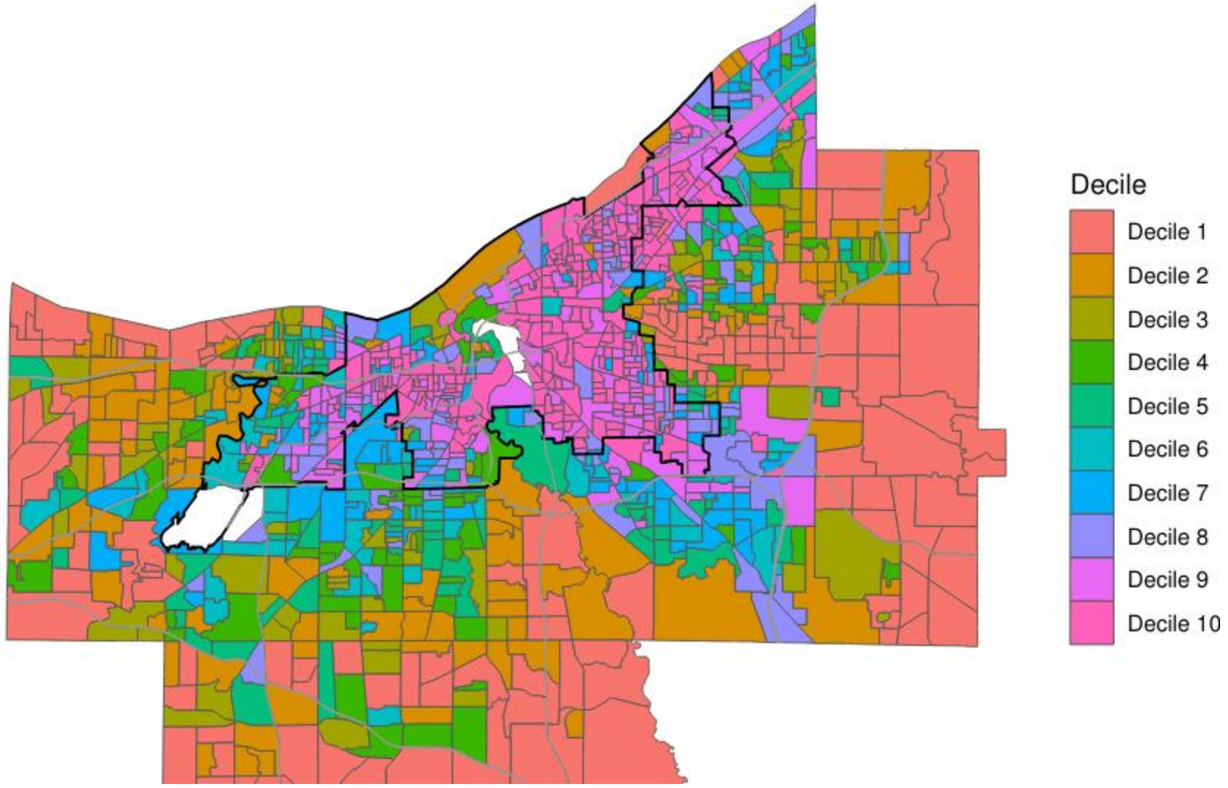

© 2024 Blazel MM et al. *JAMA Network Open*.

B) Neighborhood Atlas

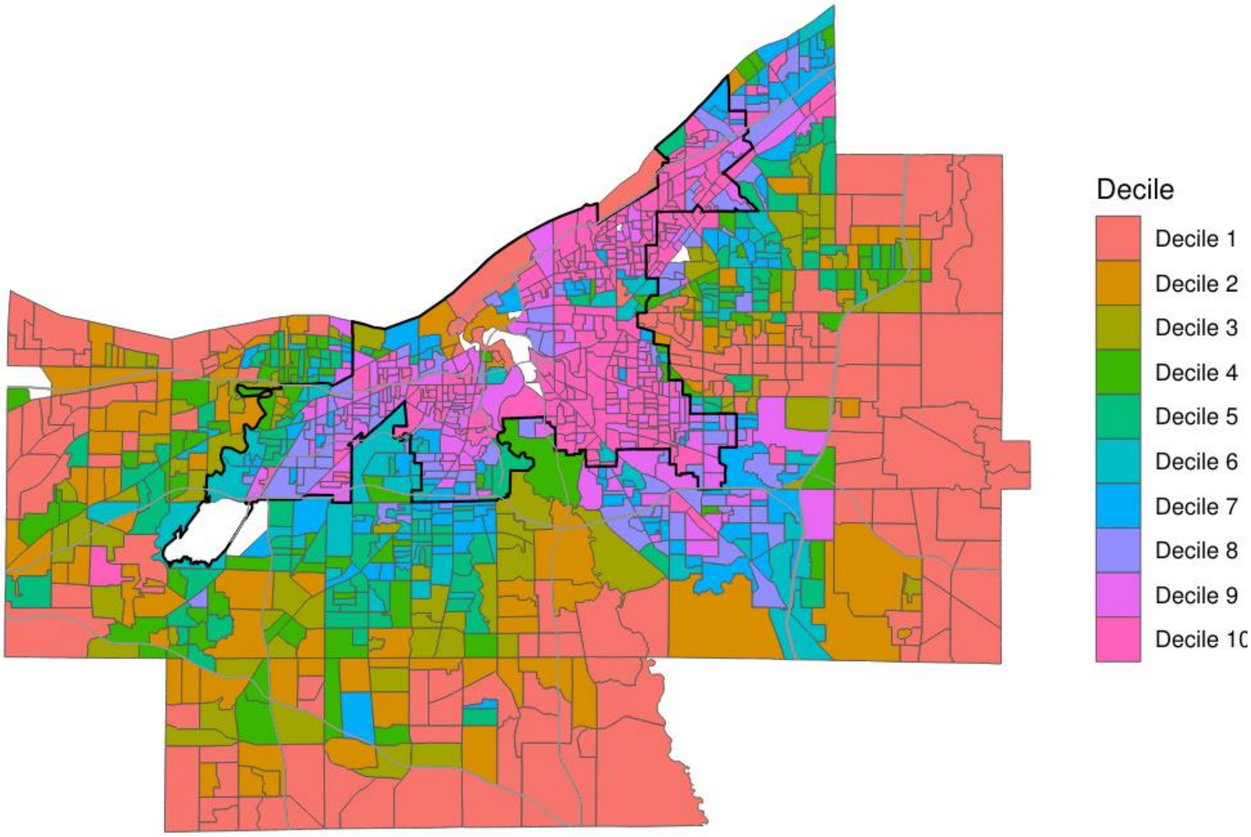

© 2024 Blazel MM et al. *JAMA Network Open*.
