# Supplementary material for: Neighborhood-Level Disparities in Hypertension Prevalence and Treatment Among Middle-Aged Adults
Source: JAMA Netw Open. 2024 Aug 23;7(8):e2429764. doi: 10.1001/jamanetworkopen.2024.29764 (PMC11344236; doi:10.1001/jamanetworkopen.2024.29764)
Supplement: Supplement 2. — Data Sharing Statement [file jamanetwopen-e2429764-s002.pdf]

Data Sharing Statement

Blazel. Neighborhood-Level Disparities in Hypertension Prevalence and Treatment Among Middle-Aged Adults. *JAMA Netw Open*. Published August 23, 2024.
doi:10.1001/jamanetworkopen.2024.29764

Data
Data available: No

Additional Information
Explanation for why data not available: The data analyzed are derived from electronic medical records, which are restricted for privacy reasons.
